# Address on the Extraction of Teeth

**Published:** 1849-10

**Authors:** J. Taft


### ADDRESS ON THE EXTRACTION OF TEETH,

READ BEFORE THE MISSISSIPPI VALLEY ASSOCIATION OF DENTAL SURGEONS AT THE FIFTH ANNUAL MEETING, HELD IN LOUISVILLE, KENTUCKY, SECOND TUESDAY OF SEPTEMBER, 1849, BY DR. J. TAFT.

Mr. President and Gentlemen: Having been appointed by this Association at its last annual meeting to prepare an Essay upon Extraction of Teeth. I have endeavored in some measure to fulfil that appointment; though circumstances over which I could exercise but little or no control, intervened and so nearly prevented the discharge of this duty, that until a few days since I had abandoned the intention of its performance. All that I will now attempt to do, will be to make some practical observations and remarks upon extraction of teeth. This operation is one of more than minor importance. It is an operation to which sometimes are attached almost insurmountable difficulties—difficulties which are overcome by but comparatively few of those who attempt the performance of this operation; difficulties which require the exercise of a fertile genius and a well disciplined judgment, to control. The fact, that this operation is so frequently called for, is a consideration worthy of note. There is no surgical operation more frequently brought into exercise than this one. Almost every person has, at some time or other, been brought to the direful necessity of submitting to it; and it is important that any thing that must be expected so often, should be done in the best possible manner. The agonizing pain attendant upon this operation, and especially when improperly performed; the accidents that are liable to follow, and every day do follow this operation, viz.: breaking the teeth off, extracting the wrong tooth, removing two or three teeth at one effort, fracture of the alveolus, dislocation of the jaw, excessive hemorrhage, &c., characterize it one of importance. The excessive dread of those about to undergo this operation, is an index not to be disregarded, when we would properly and fully view this subject. If the above is true, it is altogether necessary to be perfectly acquainted with the indications, for the exercise of this operation; if the proper indications are misapprehended, and an operation based upon that misapprehension, irremediable injuries will follow.

The following indications may be noticed:

The temporary should be removed if they are not cast at or near the time their respective permanent teeth should make their appearance through the gum. A crowded condition of the permanent with the temporary teeth, may indicate the removal of one or more of the latter. The temporary teeth are frequently subject to decay, and by this means the pulp is exposed, inflamed and diseased, and consequent upon this condition, arises some times an obstinate odontalgic which is difficult to control by the application of the ordinary innocent preparations. Hence it is proper to extract such a tooth, rather than undergo the suffering consequent upon its retention in the mouth in this condition. In all cases where the temporary teeth become the cause of inflammation and suppuration in the contiguous parts, either the periosteum alveolus or gums, a prominent indication exists for their extraction.

The indications for the extraction of the permanent teeth, are not at all times so fully manifest.

Odontalgia is an index, generally, for extraction; and especially odontalgia in its more advanced stage, is an index not to be mistaken. It is some times possible, by proper treatment, and well executed operations, to restore to health and usefulness, teeth that have but recently become painful, the pulp but slightly exposed, and the inflammation not excessive; this, combined with other favorable circumstances and a good constitution, we may anticipate a happy result without extraction. But if the pain is intense, the pulp or nerve exposed by a large orifice, inflammation excessive, and an evident tendency to suppuration, or fungus growth of the nerve, or any other diseased condition of it that could not be successfully controlled, we have a prominent index for the treatment, which is the removal of the tooth. Inflammation, and particularly chronic inflammation of the investing membrane, as a general rule, is an indication for extraction. Suppuration and ulceration of the investing membrane, clearly point to extraction as the only proper remedial treatment. It is, as a general rule, proper to remove supernumerary teeth. It is sometimes necessary to remove a sound and healthy tooth, from the regular arch, to relieve a crowded condition of the teeth, consequent upon a disproportion between the size of the teeth, and the circle in which they are set. It would be proper to remove a bicuspid or first molar, though slightly decayed, and not painful, when the teeth are rather crowded than otherwise, and where its extension in the mouth would be productive ultimately of more evil than good; this would only be indicated in persons of minority. A molar tooth having lost its antagonist, becomes elongated and consequently a source of irritation, and should be removed; yet it must be remembered, that a molar tooth cannot become elongated until two teeth are removed from the opposite jaw, for in the perfect set each molar tooth antagonizes with the one-half for the two teeth of the opposite jaw; so that this indication can only exist when two or more contiguous teeth are gone in the opposite jaw. All dead and extraneous teeth, and portions of teeth, should be removed.

Having briefly referred to some conditions that require the exercise of the operation of extraction, we will now notice more particularly the operation.

The operation under consideration, is one that demands every facility for its prompt and efficient extraction. The instruments employed in the performance of this operation, until within quite a recent period, were very ill adapted to the purpose for which they are used. The recent improvements upon this class of surgical instruments, is a subject of much congratulation to the operators of the present day; and yet they are not all in every respect that could be desired. I doubt not that the best operators of whom we can boast, sometimes, though seldom it may be, experience a want, the supplying of which would give pleasure; there are others who more sensibly and more often experience that want, without even an anticipation of ever having it supplied. Exceedingly various have been the instruments employed in this operation. The key, forceps, hook, punch, elevator, and screw, with their innumerable modifications, constitute the array of extracting instruments from time immemorial. Each of these instruments, and every modification of each instrument, has had its zealous advocate; each one, either through interest, *supposed* superiority, in real merit, extolling his favorite above all others; and frequently has real merit been kept in the back ground by this means. In this operation, the forceps have superceded almost all the other instruments that were so extensively used; and with their present modifications, a great majority of the appliances once used for this operation have become useless. The forceps are more efficient, and applicable to a greater variety of circumstances, than any other class of instruments.

The key, with its various modifications, has been employed in this operation more than any other instrument. In all the variety of modifications through which this instrument has passed, its principle of action has ever remained the same. I hope the universality of its use has not rendered it criminal to abandon its employment in the operation of extraction. There are objections to the key, which the enterprising have ever endeavored to remedy, and as often failed of success. The most prominent objections to the key are the following:—Its embrace or seizure of the tooth is altogether deficient, being made only upon one side, and with two or three sharp points; the other point of power above and at the opposite side of the tooth; a consequence of this is, that the tooth is dragged out at an angle of from fifty to ninety degrees with its axis. The fulcrum resting upon the gum and alveolus, frequently bruises, lacerates and cuts parts that should remain untouched. There are other objections to this instrument, which I will not now notice.

We are subject to less difficulties and trouble, in the employment of well constructed forceps, than any other instrument. The ill construction and adaptation of the forceps, is the only real objection that has ever existed to their use. The liability of the forceps to crush the tooth, is an objection often urged against them. This can only be predicated of those unsightly, ill constructed things which some have been pleased to denominated "*tooth nippers;*" quite an emphatic name.

There is no case in which a tooth can be extracted with the key, that could not be better done with forceps. The superiority of the forceps consists in the following things: the perfection of their embrace, the seizure extending usually to two thirds or three fourths the circumference of the tooth, and this embrace can be made farther upon the tooth, and consequently a better command obtained with the forceps than any other instrument. No serious injury is necessarily sustained by the contiguous parts, under the action of this instrument. The pain is less severe in extraction with the forceps, than with either the key or lever. In the extraction of roots, I prefer the forceps constructed for that purpose, accompanied by the compound hook, which is the hook and punch upon the same shaft, and screw. I have not as yet used any instrument upon the principle of a perfect lever. I use the compound hook in the extraction of roots more than any other instrument. The screw is an invaluable instrument, and its modifications should not escape the notice of any. It is impossible to pursue a uniformity of execution in the extraction of teeth of the same class, much less a uniformity of execution in the extraction of the different classes. The incisors and cuspidati, ordinarily, will admit of a rotary motion sufficient to break up their attachment; yet we sometimes find them in a condition that will not admit a rotary motion; then a posterior and anterior motion only is admissible, and sometimes only an anterior can with safety be applied. In the extraction of the bicuspids, can ordinarily only be applied inward and outward movement, as rotary motion is seldom admissible, and should never be attempted by any one who has not perfect confidence in his own judgment; with the first bicuspid, the point of which commonly bifurcates, it is unsafe to attempt a rotary motion at any time; the fang of the second bicuspid does not usually bifurcate. The foregoing remark is applicable only to the superior bicuspids; the fangs of the inferior are commonly cylindrical; but they do not admit of a rotary movement in their extraction. The first and second superior molars are removed with an outward and inward application of force; the first principal effort should be outward, as the palatine part of those teeth yield to the force much more readily than the labial. This is a consequence for their form and position in the jaw. The first and second inferior molars are, like the superior, susceptible only of an inward and outward motion in their extraction. They sometimes yield more readily to an outward movement, in other cases more readily to an inward; though to the former more frequently. This susceptibility can be far more easily ascertained in the use of the forceps than any other instrument. The third molars, both superior and inferior, in addition to the inward and outward movement, can in some cases be more easily removed by a backward direction of the force; these teeth commonly have but one conical fang; there is no uniform inclination in the fangs of these teeth, and their extraction should be governed by this inclination; their fangs sometimes have a posterior curvature. It is better to accommodate this inclination with a posterior movement in extraction.

In the extraction of roots, the cases are so various that it is difficult to give a uniform method of execution. There are a variety of conditions which make this a difficult operation; the unfavorable position of the roots in the jaw, the advanced stage of decay in which we sometimes find them. Roots which present the greatest difficulty are those from which the crowns have recently been broken off, and particularly those of the first and second superior molars. These can frequently be removed with the forceps constructed for that purpose; and they sometimes almost bid defiance to every effort for their removal. There are two or three methods in which an operation in an extreme case of this kind may be performed. The fangs may be separated with a separating forceps, and removed singly; or, with the lancet and knife, remove a portion of the gum and external wall of the alveolus sufficient to expose the root, so that it may be seized with the forceps, or caught with the hook and removed; it is seldom that the internal or palatine wall of the alveolus is an obstruction to an operation of this kind. Accidents sometimes attend the operation of extraction, which the most prudent cannot foresee, nor the most skilful avoid. I can now only name some of these. Alveolar hæmorrhage is an accident that in some cases attends this operation; it is occasionally of an alarming character. Fracture of the alveolus will sometimes result with the skilful; and more often, and to a greater extent, with the unskilful. Luxation of a sound tooth sometimes occurs. The breaking off of a divergent tang cannot always be avoided. Other unpleasant circumstances are liable to occur, which it should ever be the studious regard of the operator to avoid.

